# Urinary neurotransmitter analysis and canine behavior assessment

**DOI:** 10.3389/fvets.2023.1124231

**Published:** 2023-02-06

**Authors:** Teresa Schmidt, Sebastian Meller, Steven Roger Talbot, Rowena Mary Anne Packer, Holger Andreas Volk

**Affiliations:** ^1^Department of Small Animal Medicine and Surgery, University of Veterinary Medicine, Hannover, Germany; ^2^Centre for Systems Neuroscience, University of Veterinary Medicine Hannover, Hannover, Germany; ^3^Hannover Medical School, Institute for Laboratory Animal Science, Hannover, Germany; ^4^Department of Clinical Science and Services, Royal Veterinary College, Hatfield, United Kingdom

**Keywords:** neurotransmitter, behavior, behavioral problems, questionnaire, C-BARQ, urinary, canine

## Abstract

Behavioral problems are highly prevalent in domestic dogs, negatively affecting the quality of life of dogs and their owners. In humans and dogs, neuropsychological or neurobehavioral disorders can be associated with deviations in various neurotransmitter systems. Previous evidence has revealed correlations between urinary neurotransmitters and various behavioral disorders; however, a causal relationship has not yet been conclusively demonstrated. Non-invasive urinary neurotransmitter analysis may identify specific biomarkers, which enable a more differentiated assessment of canine behavioral disorders in the future and contribute to more effective neuromodulatory treatment decisions and monitoring. This approach could offer new insights into underlying pathomechanisms of canine neurobehavioral disorders. This study assessed urinary neurotransmitter levels and the descriptive behavior profile of 100 dogs using established rating scales (Canine Behavioral Assessment and Research Questionnaire, Attention Deficit Hyperactivity Disorder Rating Scale, Dog Personality Questionnaire, Canine Cognitive Dysfunction Rating Scale), and explored relationships between these variables. No correlation was found between urinary neurotransmitters and the assessed behavior profiles; however, age-, sex- and neuter-related influences were identified. The lack of correlation could be explained by the many confounding factors influencing both behavior and urinary neurotransmitter excretion, including age, sex and neuter status effects, and methodological issues e.g., low discriminatory power between anxiety and aggression in the descriptive behavior evaluation. Urinary neurotransmitter testing could not be validated as a tool for canine behavior evaluation in this study. However, reliable assessment methods with low susceptibility to human biases could be valuable in the future to support behavioral-phenotype diagnoses.

## 1. Introduction

Behavioral problems are a common concern in domestic dogs, estimated to affect 72.5–84.5 per cent of dogs (1, 2). Behaviors that owners consider “problematic” varies widely between owners, but includes a range of presentations: aggression (e.g., to owners or other dogs), anxiety (e.g., in specific contexts such as being left alone) and hyperactivity (e.g., attention-deficit/hyperactivity disorder (ADHD)-like behavior) (1, 3, 4). Dogs are highly social animals and their lives are often interwoven with their owners' daily lives. Thus, behavioral issues negatively affect the quality of life of not only affected dogs but also of their owners (5, 6). In many cases behavioral problems threaten quality and quantity of life, by contributing to owner decisions to relinquish their dog or behavioral euthanasia at a young age (1, 7–9). Problematic behavior is routinely assessed *via* patient's history, ruling out or considering medical causes as a contributing factor, and a veterinarian and/or clinical behaviorist's on-site evaluation (10–13).

In research settings, validated behavioral questionnaires completed by canine caregivers, or behavioral tests are commonly used to assess canine behavior (14–17).

To investigate neuropsychological conditions in humans, in addition to traditional psychometric scales, evidence emphasizes measuring urinary neurotransmitters and their potential usage as biomarkers (18, 19). Previous studies have found increases (dopamine, glutamate, tryptophan, serotonin) and decreases (norepinephrine, γ-aminobutyric acid [GABA]) in certain neurotransmitters in the urine of autistic children compared to healthy controls (20–23). In another investigation, elevated urinary epinephrine levels after trauma in children correlated with developing symptoms of acute posttraumatic stress disorder (24). ADHD symptoms in children and adults were linked to shifts in norepinephrine and epinephrine urinary excretion, as well as diminished phenylethylamine urine levels (25–28). Depression and anxiety symptoms were associated with enhanced urinary norepinephrine and epinephrine concentrations (29–31). Moreover, urinary dopamine metabolites strongly correlated with suicidality in depression, outperforming the cerebrospinal fluid assessment (32). For clinical application, urinary neurotransmitter analysis is commercially available in human medicine. It can assist clinicians in the differentiated workup of neuropsychological diseases, as well as selecting and monitoring effective treatment strategies (25, 33–37). However, despite the correlations indicated in multiple studies, a causal relationship between urinary neurotransmitters and the diagnosis of mental health issues has not yet been conclusively demonstrated (18). So far, urinary neurotransmitter testing is only diagnostic for a single condition, pheochromocytoma, an adrenal tumor associated with increased urinary norepinephrine (38, 39).

In veterinary medicine, biomarkers for canine behavioral and neurological disorders were also investigated in former non-targeted metabolic screenings and more targeted research approaches (37, 40–44). Tryptophan and lipid metabolites in the blood were found to correlate with canine ADHD-like behavior (40). Enhanced blood levels of glutamine and γ-glutamyl glutamine, as well as differences in molecules implicated in oxidative stress, tryptophan and lipid metabolism were detected in fearful dogs (41, 42). A low serotonin serum concentration was identified in aggressive dogs (43). A recent exploratory study focusing on urinary neurotransmitter analysis, compared samples of kennel boarding dogs with pet dogs kept in their homes (44). Increased norepinephrine and dopamine levels were identified in the kennel boarding dogs, presumably caused by stress exposure (44). Another study demonstrated that urinary neurotransmitter testing could also serve as a beneficial tool in canine epilepsy (37). Significant differences in the excretion of certain urinary neurotransmitters (glycine, serotonin, norepinephrine/epinephrine ratio, GABA/glutamate ratio) were identified between dogs affected by idiopathic epilepsy and healthy controls (37). Presented evidence corroborates correlations between neurobehavioral diseases and neurotransmitters/metabolites in the blood and urine of dogs, similar to previous findings in humans.

To date, serum serotonin analysis is commercially available to support the management of canine behavioral problems associated with fear or aggression (45). Further expansion of neurotransmitter analysis could offer new insights in certain pathway disturbances and underlying pathomechanisms of canine neurobehavioral diseases. Non-invasive urinary neurotransmitter analysis may identify specific biomarkers, which enable a more differentiated assessment of canine behavioral disorders in the future and contribute to more effective neuromodulatory treatment decisions and monitoring. The objective of this study was to evaluate whether characteristic urinary neurotransmitter deviations correlate with questionnaire assessed canine behavioral profiles.

## 2. Materials and methods

Urine samples and behavioral data from 100 privately owned dogs were collected at the University of Veterinary Medicine Hannover, Germany (TiHo) between January and June 2020. Recruited dogs were owned by TiHo staff and students, which were contacted *via* email. All owners were informed and gave consent. Included dogs were at least 6 months of age, of any breed and both sexes, and without chronic diseases or drug treatment. Dogs with owner-reported behavioral problems, as well as dogs with unremarkable behavior were included; however, given the threshold for the presence or absence of “problem behavior” likely differs between owners, detailed behavioral analysis exploring individual owner-reported behaviors without asking if they were problematic to the owner and/or their household was conducted by the below listed, formerly validated questionnaires. Urine samples were collected by the owners *via* the non-invasive free catch method. For the urinary neurotransmitter analysis, morning urine from the first or second void of the fasting dog was used. Milk products, fruit and vegetables were avoided for 48 h and strenuous exercise was avoided for 24 h before sampling. During the collection process entire females were not in heat. Samples were preserved in prepared tubes containing 50 mg oxalic acid for 10 ml urine and were immediately transferred to the TiHo laboratory. There, aliquots were stored continuously frozen at −80°C until their shipment on dry ice for external neurotransmitter analysis.

Nine urinary neurotransmitters (serotonin, histamine, glycine, phenylethylamine, dopamine, epinephrine, norepinephrine, glutamate, GABA) were quantified utilizing high-performance liquid chromatography triple-quadrupole mass spectrometry/mass spectrometry technology (Doctor's Data, St. Charles, IL, USA). Urinary creatinine levels were assessed *via* enzymatic colorimetric–kinetic Jaffé method (46). They served as a reference factor to calculate urine density and determine neurotransmitter levels relative to creatinine concentration. Usually, neurotransmitter levels in human urine samples are analyzed with the applied technology. However, recently multiple studies investigating urinary neurotransmitter concentrations have been carried out in dogs also identifying biologically reasonable results (37, 47–49).

Descriptive behavioral data of the dogs as continuous variables were gained *via* a web-based standardized owner questionnaire, hosted on LimeSurvey (LimeSurvey GmbH, Hamburg, Germany) and based on four previously validated canine behavioral questionnaires exploring a range of behavioral presentations in dogs {Canine Behavioral Assessment and Research Questionnaire [C-BARQ] (14), Attention Deficit Hyperactivity Disorder Rating Scale [ADHD RS] (15), Dog Personality Questionnaire [DPQ] (50), and Canine Cognitive Dysfunction Rating Scale [CCDR] (51)}.

## 3. Results

A total of 100 dogs were included in the study, of which 42 were males (neutered: 62 percent) and 58 females (neutered: 64 percent). The mean age was 5.35 (+/–SD 3.87) years, the mean weight was 17.03 (+/-SD 9.21) kg, and the ratio of purebred to crossbred dogs was 60:40.

Comparing the urinary neurotransmitter data revealed no correlation between neurotransmitters and the behavioral profile from any of the four canine questionnaires. A multivariate analysis of variance (MANOVA) was used to compare the multiple sample means of the nine neurotransmitters as a function of sex/neuter status and age and their interaction. The analysis indicated a significant effect concerning the main effects of sex/neuter status (*p* ≤ 0.001), age (*p* ≤ 0.001), as well as their interaction (*p* ≤ 0.02) on urinary neurotransmitter excretion. Since all nine neurotransmitters had a significant effect, the effect on particular neurotransmitters was subsequently determined with follow-up analyses of variance (ANOVA). Here, the neurotransmitters were set as dependent variables, with sex/neuter status and age and their interaction as independent variables. The results of the ANOVAs were adjusted for multiple testing using the Bonferroni criterion. The analyses identified an effect of the sex and neuter status on the urinary concentration of histamine (*p* ≤ 0.05), norepinephrine (*p* ≤ 0.05), phenylethylamine (*p* ≤ 0.05) and dopamine (*p* ≤ 0.001). Age significantly affected the urinary levels of norepinephrine (*p* ≤ 0.01) and dopamine (*p* ≤ 0.001).

**Figure 1:**
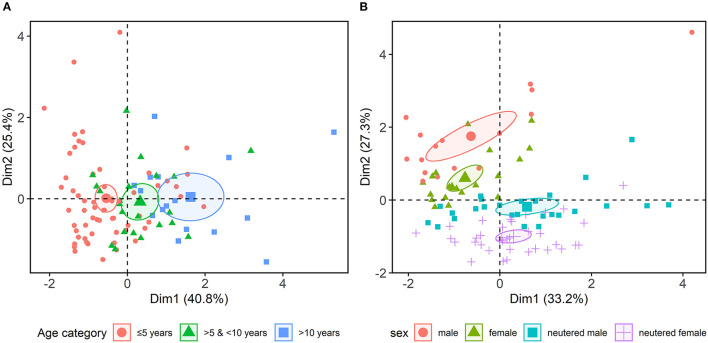
Combined principal component analysis, focussing on age, sex, neutering and the three most significant neurotransmitter variables (norepinephrine, dopamine, γ-aminobutyric acid/glutamate ratio) revealed **(A)** age- and **(B)** age sex/neuter-dependent clusters, indicating a hormonal influence on urinary neurotransmitters excretion.

Principal component analysis (PCA) was used to reduce the complexity of multiple variables (age, sex) and put them into context with the three most significant neurotransmitters (norepinephrine, dopamine, GABA/glutamate ratio). The linear combinations of all variables were projected into two dimensions, preserving most of the variance information (60.5%) of the original data. The projections visualize age- and sex/neuter-dependent clusters of the excreted neurotransmitters as 95% confidence ellipses (Figure 1).

**Figure 2:**
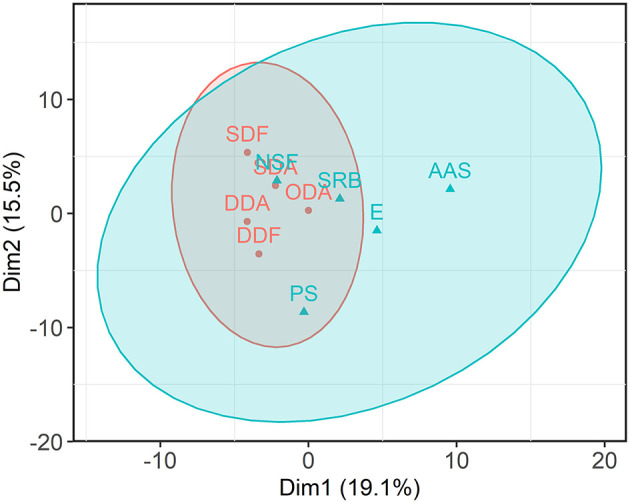
Cleaned cluster analysis showed clustering of Canine Behavioral Assessment and Research Questionnaire (C-BARQ) factors reflecting social fear and aggression (dog-directed fear [DDF], dog-directed aggression [DDA], stranger-directed fear [SDF], stranger-directed aggression [SDA], owner-directed aggression [ODA]) and another clustering of mixed factors (excitability [E], attachment or attention-seeking [AAS], separation-related behavior [SRB], nonsocial fear [NSF], pain sensitivity [PS]), when non correlating C-BARQ factors (chasing, trainability) were previously excluded, indicating a low discriminatory power of fear and aggression.

Further, 11 behavioral C-BARQ factors were *z*-transformed from multiple raters and analyzed in a PCA. Due to the variance in the original data, the projections in the first two dimensions captured 34.6% of the information. A subsequent cluster analysis with the *k*-means algorithm revealed two formations (indicated with 95% confidence ellipses in Figure 2). When non-correlating C-BARQ factors (chasing, trainability) were excluded, the first cluster covered factors representing social fear and aggression behavior (dog-directed fear, dog-directed aggression, stranger-directed fear, stranger-directed aggression, and owner-directed aggression). The second cluster included excitability, attachment or attention-seeking, separation-related behavior, nonsocial fear, and pain sensitivity. No significant results were found in the other questionnaires (ADHD RS, DPQ, CCDR).

## 4. Discussion

The present study investigated whether urinary neurotransmitter deviations correlate with descriptive canine behavioral profiles. Evidence in human medicine has previously revealed a correlation between urinary neurotransmitters and neuropsychological disorders (18, 19). In veterinary medicine, blood screenings in dogs also showed an association between neurotransmitters/metabolites and neurobehavioral diseases (40–43). Limited pioneering studies in dogs, indicated non-invasive assessment of urinary neurotransmitters and their usage as potential biomarkers of stress and epilepsy (37, 44), which might be also a valuable approach to canine behavioral health care. In the current study no significant correlations between urinary neurotransmitter levels and canine behavioral profiles were found. However, an effect of age, sex and neuter status on canine urinary neurotransmitter excretion demonstrated in this study, indicates a hormonal influence (Figure 1). These findings are consistent with those of human studies, showing age- and sex-related variations in urinary neurotransmitter excretion of dopamine, epinephrine and norepinephrine (52, 53). Sex differences in the urinary neurotransmitter profile were assumed to be caused by associated differences in behavioral patterns of both sexes in humans, which could not be corroborated for dogs in this study (53).

Although, urinary neurotransmitter deviations have been previously associated with different neuropsychological conditions in humans, those results were not transferable to the investigated canine population, and its value for canine behavioral medicine could not be confirmed from these results. This lack of conformity is following some earlier serum serotonin level studies and may be explained by the fact that a direct correlation between the central nervous system (CNS) and the urinary neurotransmitter levels only have been shown to a limited extent in previous studies (18, 19, 54, 55). During neurotransmitter transfer through the body, transporters of the blood-brain barrier (BBB) and the kidney modulate their concentration, whereby the BBB is not permeable for every neurotransmitter (18). In addition to the CNS synthesis, neurotransmitters are also produced in the peripheral nervous system, the gut microbiome, in most body organs and can be influenced by nutrition (56–63). To minimize potential bias, solely healthy dogs without organ diseases and normal renal function were included in the current study. Furthermore, certain nutrients classified as external neurotransmitter or precursor sources were restricted before sample acquisition (62, 64, 65). A recent study was able to demonstrate a correlation of serine, glycine and norepinephrine levels between the cerebrospinal fluid, blood and urine of dogs, indicating an association of the CNS and peripheral neurotransmitters (47). Despite internal and external influences, previous investigations of canine urinary neurotransmitters found associations to stress exposure and epilepsy (37, 44). Therefore, urinary neurotransmitter analysis might be used as adjunctive screening tool for stress responses or other canine neurological diseases in the future.

The C-BARQ scores were affected by an age-related sex and neuter status effect. Intact dogs of both sexes had higher overall C-BARQ scores with advanced age, indicating a hormonal influence (Figure 2). This accords with earlier studies that found variables such as breed, sex and neuter status significantly affecting the C-BARQ scores (66–70). In addition, the exposure period to gonadal hormones in females has been negatively correlated with fear and aggression scores (67).

Cleaned cluster analysis showed clustering of C-BARQ factors reflecting social fear and aggression and another clustering of mixed behavior factors (Figure 2). These findings imply that if analyzed as an overall score, the C-BARQ primarily reflects anxiety and fearful behavior, with low discriminatory power of fear and aggression using the statistical approach applied in the investigated study population. This seems to be consistent with other studies. For example, in a Portuguese population, dog-directed fear and aggression factors were overlapping and in a study from Japan, fear and aggression items were similarly merged (71, 72). The overlap might be biologically driven e.g., fear being the emotional state underlying aggressive behavioral responses in some individual dogs, or may reflect owners' lack of ability to interpret and report dog behavior (73). Regional differences are considered another reason, and a comparison of matching groups on an international level has been recommended (66, 72).

A main limitation of the study is behavior being exclusively assessment *via* owner-reported questionnaires. Such data may involve owner related bias, due to the subjective interpretation of their dogs' behavior, understanding of the terminology in each questionnaire, and potentially social desirability effects. Therefore, it must be considered that the actual behavior profile of the dogs might not have been reflected in the analyses. Behavior is complex and so are behavioral disorders (74); in contrast, current behavior classification systems reduce the many facets of behavior to simpler descriptive terms e.g., aggression (74). More in-depth behavioral investigations utilizing multi-axis models may offer a valuable option for behavioral analysis in the future (74).

Behavior assessment of dogs presenting with behavioral problems remains a challenging task and is subject to the subjective biases of human interpretation (e.g., the knowledge and experience of the assessor). Urinary neurotransmitter analysis has the potential to serve as a novel, objective complement in canine behavior evaluation, especially for longitudinal studies; however, the tool could not be validated in this study population (75). Nevertheless, urinary neurotransmitter testing deserves further studies in more severely affected populations e.g., studies of specific behavioral cases diagnosed in a behavioral clinic vs. behaviorally healthy controls matched on influential factors such as age, sex and neuter status, to better understand their potential role in canine behavior assessment.

## Data availability statement

The raw data supporting the conclusions of this article will be made available by the authors, without undue reservation.

## Ethics statement

Collection of urine samples was performed using the non-invasive free catch method and did not require ethical approval. Descriptive behavioral data of the dogs were gained *via* a web-based standardized owner questionnaire. The study was conducted following the guidelines of the University of Veterinary Medicine Hannover and approved by the Thesis Committee of the University. Written informed consent was obtained from the owners for the participation of their animals in this study.

## Author contributions

TS participated in the planning of the study, carried out the main practical work, the recruitment, the sample acquisition, and drafted the manuscript. HV designed and coordinated the study. SM supported sample acquisition. SM and HV made essential contributions to the conception and acquisition of data. ST performed the statistical analysis and wrote sections of the manuscript. SM, ST, RP, and HV critically reviewed and edited the manuscript for important intellectual content. All authors contributed to the manuscript revision, read, and approved the final manuscript.
